# Multidimensional Optimization of Signal Space Distance Parameters in WLAN Positioning

**DOI:** 10.1155/2014/986061

**Published:** 2014-03-16

**Authors:** Milenko Brković, Mirjana Simić

**Affiliations:** School of Electrical Engineering, University of Belgrade, Bulevar kralja Aleksandra 73, 11120 Belgrade, Serbia

## Abstract

Accurate indoor localization of mobile users is one of the challenging problems of the last decade. Besides delivering high speed Internet, Wireless Local Area Network (WLAN) can be used as an effective indoor positioning system, being competitive both in terms of accuracy and cost. Among the localization algorithms, nearest neighbor fingerprinting algorithms based on Received Signal Strength (RSS) parameter have been extensively studied as an inexpensive solution for delivering indoor Location Based Services (LBS). In this paper, we propose the optimization of the signal space distance parameters in order to improve precision of WLAN indoor positioning, based on nearest neighbor fingerprinting algorithms. Experiments in a real WLAN environment indicate that proposed optimization leads to substantial improvements of the localization accuracy. Our approach is conceptually simple, is easy to implement, and does not require any additional hardware.

## 1. Introduction

Possession of the information about user location in radio networks, where the user mobility is assumed, completes the goal of service availability, not only at the right time but also in the right place. The idea of user positioning in radio networks originated in cellular networks for safety purposes. Emergency call services (such as 911 and 112) are the best example, since users are not always able to provide precise information of their location to emergency dispatchers. This is the main reason why regulatory agencies in the USA and the EU required mobile network operators to implement location services in their networks. Later, the information about mobile user location opened new commercial possibilities for mobile network operators. A large number of applications in radio networks based on user location information in outdoor environment initiated development of similar applications for indoor environment. Some of the indoor LBS are inventory tracking, location detection of products stored in a warehouse, location detection of medical personnel or equipment in a hospital, location detection of firemen in a building on fire, virtual tourist guides in museums and galleries, and so forth [1]. Although the indoor positioning implies positioning in various short-range technologies [2] as bluetooth, RFID (Radio Frequency IDentification), IrDA (Infrared Data Association), UWB (Ultra Wide Band), and so forth, WLAN is the most common technology in indoor environment, and therefore is used for indoor positioning the most often.

Regardless of the type of radio technology, there are four signal parameters which can be used for user positioning in radio networks [3]: AOA (Angle Of Arrival), TOA (Time Of Arrival), TDOA (Time Difference Of Arrival), and RSS. AOA parameter is very sensitive to the NLOS (Non-Line-Of-Sight) propagation conditions and multiple propagation, being the main characteristics of indoor propagation [4]. Also, AOA requires considerable hardware modifications on the transmitter/receiver, and therefore is not suitable for indoor positioning. Time as a parameter (either TOA or TDOA) is generally very reliable positioning parameter, but, like AOA, not suitable for indoor conditions. The main problem here is the demand for high resolution of time measurement. This is a consequence of the wave propagation at the speed of light over small distances inside buildings. This leaves RSS as the only parameter available to estimate the distance, according to a propagation model.

Signal propagation in indoor environments is a complex process and propagation modeling requires large number of input parameters. Building type, infrastructure, wall thickness, windows layout, and size of the rooms all have significant influence on signal propagation. Even knowledge of all these parameters cannot guarantee good estimation of the RSS, because the number and position of people moving within the building also affect the signal level. Therefore, the most challenging issue for indoor positioning based on RSS parameter is the unstable RSS value due to the multipath effect caused by reflection, diffraction, and diffusion on the indoor scattering-rich walls, which causes a time-varying RSS even at a fixed location [5]. However, despite numerous drawbacks, RSS parameter is the most common choice for positioning in indoor environment, mainly due to lack of alternative.

Complex indoor radio propagation in practice reduces the indoor positioning techniques to two: the location proximity (also known as Cell-ID) and fingerprinting [6]. Cell-ID is the simplest positioning technique based on the fact that the location of the nearest transmitter (in case of WLAN, location of the access point, AP) is assigned to an unknown user/terminal location. This method has all the advantages and only one drawback—poor accuracy. Fingerprinting technique is more complex compared to Cell-ID, but increases positioning accuracy. Location fingerprinting refers to techniques that match the fingerprint of a set of radio signal propagation characteristics that are location dependent [1].

A number of various location fingerprinting-based positioning algorithms are available: probabilistic methods [7], neural networks [8], space segmentation [9], support vector machines [10], and *K* nearest neighbor (KNN), as one of the basic algorithms [11]. KNN uses the online RSS to search for *K* closest matches of known locations in signal space from the previously built database. By averaging these *K* location candidates with or without adopting the distances in signal space as weights, an estimated location is obtained [1].

The algorithm proposed in this paper improves precision of WLAN positioning, based on nearest neighbor fingerprinting algorithms, by applying optimization of the parameters of the metric used to compute the difference in radio fingerprints. Experimental results in a real indoor WLAN environment show that the optimization of signal space distance computational parameters results in higher positioning accuracy compared to the traditional approach.

The rest of the paper is structured as follows.  Section 2 illustrates basic principles for the location fingerprinting system.  Section 3 describes the optimization of the parameters used to compute the difference in radio fingerprints. In Section 4, we describe the experimental environment.  Section 5 presents experimental results and analyses. Finally, the conclusion is given in Section 6.

## 2. Fingerprinting Positioning Algorithm

Fingerprinting is a positioning method that relies upon a database of collected location dependent radio signal parameters that constitute a “fingerprint” of a specific location. Location of the mobile unit is determined comparing a set of radio signal parameters the mobile unit observes to available database.

The first step in fingerprinting is to collect a set of location dependent parameters of radio signals received by the mobile user (MU) from the access points. The access points are assumed to have fixed spatial positions. In WLAN, the location-dependent parameter readily available is the value of the received signal strength of available access points at the considered point in space. To build the database of location fingerprints in the area of interest, a set of reference points (RPs) has to be selected. The RPs should be uniformly spread in the area of interest [12]; that is, the area in which the positioning service is being implemented. This area is also called the “test bed” area [13].

The process of collecting the database of fingerprints is named the training phase. Result of the training phase is the database containing a set of *n*_RP_ coordinates, (*x*_RP*k*_, *y*_RP*k*_), *k* ∈ {1,…  *n*_RP_}, accompanied by the corresponding ordered set of observed RSS levels that originate from all of the available access points, indexed by *i*, *i* ∈ {1,…, *n*}. The set of measured RSS levels is conveniently stored in a vector

(1) $$\begin{array}{c} {\vec{\text{RSS}}}_{\text{RP}k}=\left[\text{RS}{\text{S}}_{\text{RP}k,1},\text{RS}{\text{S}}_{\text{RP}k,2},\ldots ,\text{RS}{\text{S}}_{\text{RP}k,n}\right]. \end{array}$$

The vector ${\vec{\text{RSS}}}_{\text{RP}k}$ is a fingerprint of the location specified by (*x*_RP*k*_, *y*_RP*k*_).

After the system is trained, that is, the database of fingerprints that correspond to the RPs is collected, in the positioning phase of the algorithm for each positioning request the mobile unit measures the vector of RSS parameters it observes, ${\vec{\text{RSS}}}_{\text{MU}}$,

(2) $$\begin{array}{c} {\vec{\text{RSS}}}_{\text{MU}}=\left[\text{RS}{\text{S}}_{\text{MU},1},\text{RS}{\text{S}}_{\text{MU},2},\ldots ,\text{RS}{\text{S}}_{\text{MU},n}\right], \end{array}$$

and compares it to the database obtained in the training phase of the method. An appropriate algorithm determines location of the mobile unit which is the most likely.

### 2.1. Metric for the Difference in Radio Fingerprints

Many algorithms can be used to estimate position of the mobile unit. The basic one is the nearest neighbor in signal space (NNSS or NN) [11]. First, the signal distance between the RSS vector observed by the mobile unit (2) and the RSS vectors in the database (1) is computed. In this paper, we propose generalized signal space distance defined as

(3) $$\begin{array}{c} L_{qm}={\left(\sum_{i=1}^{n}{\left|\text{RS}{\text{S}}_{\text{RP}m,i}-\text{RS}{\text{S}}_{\text{MU},i}\right|}^{q}\right)}^{1/q}, \end{array}$$

where, 1 ≤ *m* ≤ *n*_RP_ and *n*_RP_ is the number of RPs available in the database. For *q* = 2, generalized distance of (3) reduces to the Euclidean distance, while for *q* = 1 the distance is the Manhattan distance. The metric maps a difference of RSS vectors to a single real number.

After the signal space distances from the observed RSS vector to the vectors available in the database are computed, the minimum is found:

(4) $$\begin{array}{c} L_{q\;\min}=\underset{1\leq m\leq n_{\text{RP}}}{\min}L_{qm} \end{array}$$

which determines the reference point *l* which is in the radio signal space the closest to mobile unit

(5) $$\begin{array}{c} L_{ql}=L_{q\;\min}. \end{array}$$

Coordinates of this reference point (*x*_RP*l*_, *y*_RP*l*_) are assigned as an estimate of the mobile unit coordinates

(6) $$\begin{array}{c} \left(x_{\text{MU}},y_{\text{MU}}\right)=\left(x_{\text{RP}l},y_{\text{RP}l}\right). \end{array}$$

Although heuristic in nature, the nearest neighbor search is natural and obvious. However, the positioning ultimately relies on only one RSS vector in the database. Another issue is opened in the case where more than one of the vectors in the database satisfies (5). A response to these issues is a generalized method, named *K* nearest neighbors (KNN). The algorithm determines a set *S*_NN_ of *K* reference points with the RSS vector ${\vec{\text{RSS}}}_{\text{RP}k}$ closest to the RSS vector observed by the mobile unit, ${\vec{\text{RSS}}}_{\text{MU}}$, and assigns estimate of the mobile unit position according to

(7) $$\begin{array}{c} x_{\text{MU}}=\frac{1}{K}\sum_{i=1}^{K}x_{\text{RP}\;S_{\text{NN}}\;i},\\ y_{\text{MU}}=\frac{1}{K}\sum_{i=1}^{K}y_{\text{RP}\;S_{\text{NN}}\;i}. \end{array}$$

In this manner, all of the reference points in the *S*_NN_ have the same influence in determining the mobile unit position.

To include the information about the radio distance from the mobile unit to the reference points in *S*_NN_, another algorithm to compute the estimated position of the mobile unit is proposed as

(8) $$\begin{array}{c} x_{\text{MU}}=\frac{\sum_{i=1}^{K}\left(\left(1/L_{q,S_{\text{NN}}\;i}\right)x_{\text{RP}\;S_{\text{NN}}\;i}\right)}{\sum_{i=1}^{K}\left(1/L_{q,S_{\text{NN}}\;i}\right)},\\ y_{\text{MU}}=\frac{\sum_{i=1}^{K}\left(\left(1/L_{q,S_{\text{NN}}\;i}\right)y_{\text{RP}\;S_{\text{NN}}\;i}\right)}{\sum_{i=1}^{K}\left(1/L_{q,S_{\text{NN}}\;i}\right)}. \end{array}$$

This method of estimating the mobile unit coordinates takes into account both *K* nearest neighbors, as well as their signal space distance to the mobile unit. Similar to KNN, but with the weighting scheme (inverse of the signal space distance as a weight), this method is known as *K* weighted nearest neighbors (KWNN) [14].

All three of the considered algorithms are heuristic in nature and require database of radio fingerprints observed in a set of reference points. The algorithms that include *K* nearest neighbors require somewhat more complex database search algorithm. Common to all three of the algorithms is the dependence of the positioning result on the metric used to compute the difference in radio fingerprints, characterized by the exponent *q*.

### 2.2. Received Signal Strength Value Assigned to Unavailable Access Points

Another relevant issue in the fingerprinting approach is the RSS value assigned to the access points that are effectively unobservable by the mobile unit. Since the RSS is measured in dB, absence of the signal corresponds to minus infinity, causing the difference measure (3) to diverge every time when some of the access points represented in the RSS vector are not observable. This problem is heuristically patched by assuming a low finite RSS value for the access points that are not observable. The assumed value will be labeled as *noAP*. The value chosen for *noAP* affects the result of (3), propagating further to affect the positioning result. Thus, *noAP* effectively appears as an indirect parameter of the signal space distance metric.

## 3. Multidimensional Optimization

The metric used to compute the difference in radio fingerprints, parameter *q*, usually takes a value of 1 or 2, so we have Manhattan or Euclidean distance, respectively, although there is no physical limitations referring to the values that parameter *q* could take. By varying this parameter, within a reasonable range, we will try to reduce the positioning error.

When measuring the RSS parameters at one point, it often happens that signal strength of some APs is below the threshold and the device simply does not detect a signal from the AP. The question is what RSS value should be assigned for these APs, so that numerical value of the signal space distance can be calculated? In the previous section we called this parameter *noAP*. Most commonly, this parameter is assigned value of the noise level, but we will optimize its value in order to increase positioning accuracy.

Multidimensional optimization is a process of finding optimal values of the signal space distance parameters *q* and *noAP* in order to maximize accuracy of the WLAN positioning. In this paper, we performed multidimensional optimization for all considered algorithms (NN, KNN, and KWNN) and the results are presented in Section 5.

## 4. Experimental Setup

**Figure 1:**
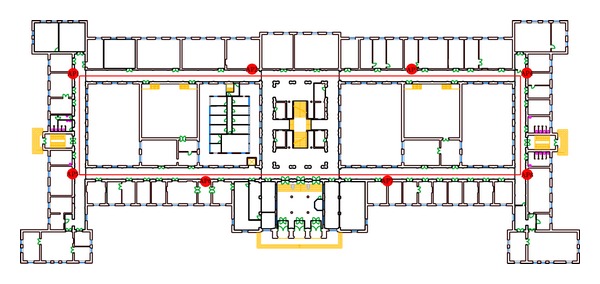
Experimental test bed.

Measurements required for the experimental analysis of fingerprinting positioning method are performed in hallways of the School of Electrical Engineering, University of Belgrade, using the WLAN available there. The experimental area is of the size 147 m × 66 m. The WLAN infrastructure consists of eight APs placed to provide the best signal coverage. The APs are Cisco Aironet 1230G high capacity with omnidirectional antennas supporting 802.11g WLAN standard on 2.4 GHz with 50 mW of power. All of the APs are placed 0.2 m beneath the ceiling. The building plan is presented in Figure 1. Positions of the APs are marked with red dots, and red rectangle shows the area where the measurements were conducted. The building is with narrow hallways and lot of walls and windows, where prediction of wave propagation is very difficult.

**Figure 2:**
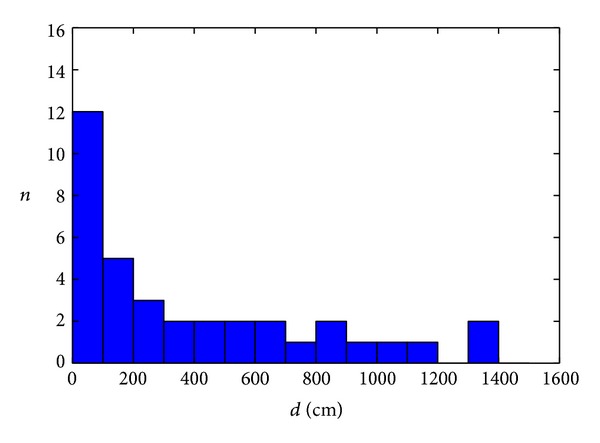
Histogram of the positioning error for NN algorithm without optimization.

Fingerprinting database is formed from RSS measurements collected at 147 RPs uniformly distributed in the middle of the hallways (red rectangle in Figure 1) and 2 m apart from each other. Thirty-seven test points (TPs) are also located on the rectangle, however, outside of strictly defined uniform schedule at every 2 m, and they are used to check accuracy of the implemented positioning method. Measurements were performed in a real workday environment, with N5010 Dell laptop and DV1501 Wireless-N WLAN Half-Mini Card network card, using inSSIDer 2.0.

## 5. Experimental and Optimization Results

The first method to be considered in this paper is the NN algorithm. The measurement results are first processed without multidimensional optimization. Mean positioning error obtained by NN algorithm, defined as

(9) $$\begin{array}{c} \delta =\frac{1}{N}\sum_{i=1}^{N}\sqrt{{\left(x_{\text{MU}}-x_{\text{MU},\text{actual}}\right)}^{2}+{\left(y_{\text{MU}}-y_{\text{MU},\text{actual}}\right)}^{2}}, \end{array}$$

is *δ* = 434.39 cm, where (*x*_MU,actual_, *y*_MU,actual_) are actual coordinates of the mobile unit and *N* is the number of test points. This value is obtained for the parameter value *noAP* = −100 dBm (equal to the receiver sensitivity) and Euclidean distance (*q* = 2).  Figure 2 shows a histogram of the positioning error for the NN algorithm.

**Figure 3:**
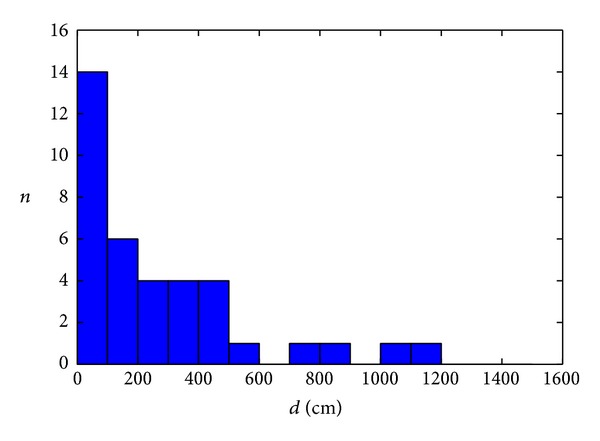
Histogram of the positioning error for NN algorithm with optimization.

**Figure 4:**
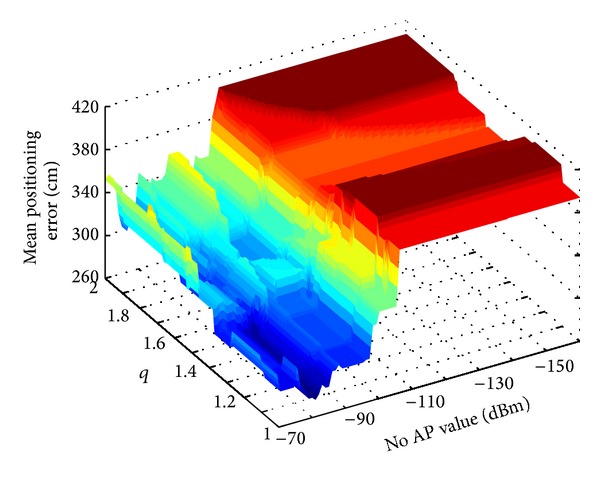
Mean positioning error dependence of the signal space distance parameters *q* and *noAP*.

Optimization of a signal space distance parameters *q* and *noAP* is performed applying the brute force algorithm, in order to obtain the minimum average positioning error. The resulting mean positioning error in the case of NN algorithm with optimization is *δ* = 270.03 cm. Optimization of the signal space distance parameters results in the mean positioning error reduction of about 40%, without any effort in the fingerprint database generation, only by applying the optimization process.  Figure 3 shows histogram of the positioning error for NN algorithm with multidimensional optimization. The obtained optimal values of the parameters are *q* = 1.1 and *noAP* = −81 dBm.  Figure 4 shows mean positioning error dependence of the positioning parameters *q* and *noAP*.

In order to evaluate the effect of optimization for KNN and KWNN algorithm, we performed the same analyses as in the case of NN algorithm. In Table 1, mean positioning error is presented. In the case without optimization, in the considered WLAN environment increase of parameter *K* slightly increases the mean positioning error.

Multidimensional optimization of parameters *q* and *noAP* is also performed for KNN and KWNN algorithms in order to minimize mean positioning error. Results shown in Table 2 show that optimization leads to a reduction in mean positioning error both for KNN and KWNN algorithm but also depends on the value of parameter *K*. The best results of multidimensional optimization are achieved for *K* = 2, where the mean positioning error is reduced by about 48% for KNN and by about 36% for KWNN algorithm, which yields from the data presented in Tables 1 and 2.

Optimal values of the optimization parameters *q* and *noAP* are different for different algorithms and different values of the parameter *K*, and they are given in Table 3.

Presented experimental results show that the most convenient positioning choice for the considered indoor WLAN environment is KNN algorithm (with *K* = 2) with optimized parameters *q* = 1.1 and *noAP* = −77 dBm. In this case, mean positioning error reaches the minimum value of *δ* = 252.89 cm. This represents maximum accuracy limit of fingerprinting-based positioning method for given indoor WLAN environment. In this manner, any indoor WLAN environment can be characterized by the set of signal space distance parameter values {*q*, *noAP*} which guarantee the highest possible indoor positioning accuracy. At this moment, it remains an open question whether there is a physical explanation for the optimal values of these parameters, or it is just an empirical result.

## 6. Conclusion

To improve precision of WLAN positioning based on traditional fingerprinting nearest-neighbor algorithms, the optimization of parameters used to compute the difference in radio fingerprints is proposed. Experiments in a real WLAN environment demonstrated usefulness of the optimization process and significant improvements of the positioning accuracy. The numerical results show reduction of the mean positioning error from about 20% to 48% for all of the considered algorithms. Furthermore, optimal values of the optimization parameters guarantee the highest possible indoor positioning accuracy and can be used as a unique positioning characteristic of any indoor WLAN environment. The approach is conceptually simple, is easy to implement, and does not require any additional hardware, time, or effort compared to traditional fingerprinting positioning process.

## Figures and Tables

**Table 1:** Mean positioning error, δ, for KNN and KWNN algorithm without optimization.

*K*	δ (cm)	
	KNN	KWNN
2	488.77	440.29
3	528.20	478.50
4	620.25	526.42
5	707.62	554.66

**Table 2:** Mean positioning error, δ, for KNN and KWNN algorithm with optimization.

*K*	δ (cm)	
	KNN	KWNN
2	252.89	283.37
3	296.93	331.17
4	376.39	376.44
5	496.48	444.14

**Table 3:** Dependance of the optimal *q* and *noAP* values on the algorithm type and parameter *K*.

*K*		*q*	*noAP* (dBm)
2	KNN	1.1	−77
	KWNN	1.1	−77

3	KNN	1.42	−87
	KWNN	2	−87

4	KNN	1.42	−87
	KWNN	2	−82

5	KNN	1.41	−87
	KWNN	2	−87

## References

[1] Liu H, Darabi H, Banerjee P, Liu J (2007). Survey of wireless indoor positioning techniques and systems. *IEEE Transactions on Systems, Man and Cybernetics C*.

[2] Bensky A (2008). *Wireless Positioning Technologies and Applications*.

[3] Melikov A (2011). *Cellular Networks: Positioning, Performance Analysis, Reliability*.

[4] Pahlavan K, Li X, Mäkelä J-P (2002). Indoor geolocation science and technology. *IEEE Communications Magazine*.

[5] Fang S-H, Lin T-N, Lee K-C (2008). A novel algorithm for multipath fingerprinting in indoor WLAN environments. *IEEE Transactions on Wireless Communications*.

[6] Sun G, Chen J, Guo W, Liu KJR (2005). Signal processing techniques in network-aided positioning: a survey of state-of-the-art positioning designs. *IEEE Signal Processing Magazine*.

[7] Roos T, Myllymäki P, Tirri H, Misikangas P, Sievänen J (2002). A probabilistic approach to WLAN user location estimation. *International Journal of Wireless Information Networks*.

[8] Battiti R, Nhat TL, Villani A (2002). Location-aware computing: a neural network model for determining location in wireless LANs. *Technical Report*.

[9] Simic M, Pejović P (2008). An algorithm for determining mobile station location based on space segmentation. *IEEE Communications Letters*.

[10] Wu Z-L, Li C-H, Ng JK-Y, Leung KRPH (2007). Location estimation via support vector regression. *IEEE Transactions on Mobile Computing*.

[11] Bahl P, Padmanabhan VN RADAR: An in-building RF-based user location and tracking system.

[12] Küpper A (2005). *Fundamentals of Positioning in Location-Based Services: Fundamentals and Operation*.

[13] Li B, Salter J, Dempster AG, Rizos C Indoor positioning techniques based on wireless LAN.

[14] Battiti R, Brunato M, Villani A (2002). Statistical learning theory for location fingerprinting in wireless LANs. *Technical Report*.

